# Appointment

**Published:** 1858-04

**Authors:** 


*Appointment.*—The Chair of Anatomy and Physiology in the Baltimore College of Dental Surgery, made vacant by the death of Professor W. R. Handy, has been filled by the appointment of A. Snowden Piggot, M. D., late Professor of Anatomy and Physiology in the Medical Department of the Washington University. Sen. Ed.

